# In Vitro 3D Cultures to Reproduce the Bone Marrow Niche

**DOI:** 10.1002/jbm4.10228

**Published:** 2019-10-01

**Authors:** Justin Ham, Lauren Lever, Maura Fox, Michaela R Reagan

**Affiliations:** ^1^ Center for Molecular Medicine Maine Medical Center Research Institute Scarborough ME USA; ^2^ University of New England Biddeford ME USA; ^3^ University of Maine Graduate School of Biomedical Science and Engineering Orono ME USA; ^4^ Sackler School of Graduate Biomedical Sciences Tufts University Boston MA USA

**Keywords:** 3D MODELS, BONE MARROW, BONE MARROW ADIPOSE, IN VITRO MODELS, TISSUE ENGINEERING

## Abstract

Over the past century, the study of biological processes in the human body has progressed from tissue culture on glass plates to complex 3D models of tissues, organs, and body systems. These dynamic 3D systems have allowed for more accurate recapitulation of human physiology and pathology, which has yielded a platform for disease study with a greater capacity to understand pathophysiology and to assess pharmaceutical treatments. Specifically, by increasing the accuracy with which the microenvironments of disease processes are modeled, the clinical manifestation of disease has been more accurately reproduced in vitro. The application of these models is crucial in all realms of medicine, but they find particular utility in diseases related to the complex bone marrow niche. Osteoblast, osteoclasts, bone marrow adipocytes, mesenchymal stem cells, and red and white blood cells represent some of cells that call the bone marrow microenvironment home. During states of malignant marrow disease, neoplastic cells migrate to and join this niche. These cancer cells both exploit and alter the niche to their benefit and to the patient's detriment. Malignant disease of the bone marrow, both primary and secondary, is a significant cause of morbidity and mortality today. Innovative study methods are necessary to improve patient outcomes. In this review, we discuss the evolution of 3D models and compare them to the preceding 2D models. With a specific focus on malignant bone marrow disease, we examine 3D models currently in use, their observed efficacy, and their potential in developing improved treatments and eventual cures. Finally, we comment on the aspects of 3D models that must be critically examined as systems continue to be optimized so that they can exert greater clinical impact in the future. © 2019 The Authors. *JBMR Plus* published by Wiley Periodicals, Inc. on behalf of American Society for Bone and Mineral Research.

## Introduction

### Bone structure and cellular makeup

**Figure 1:**
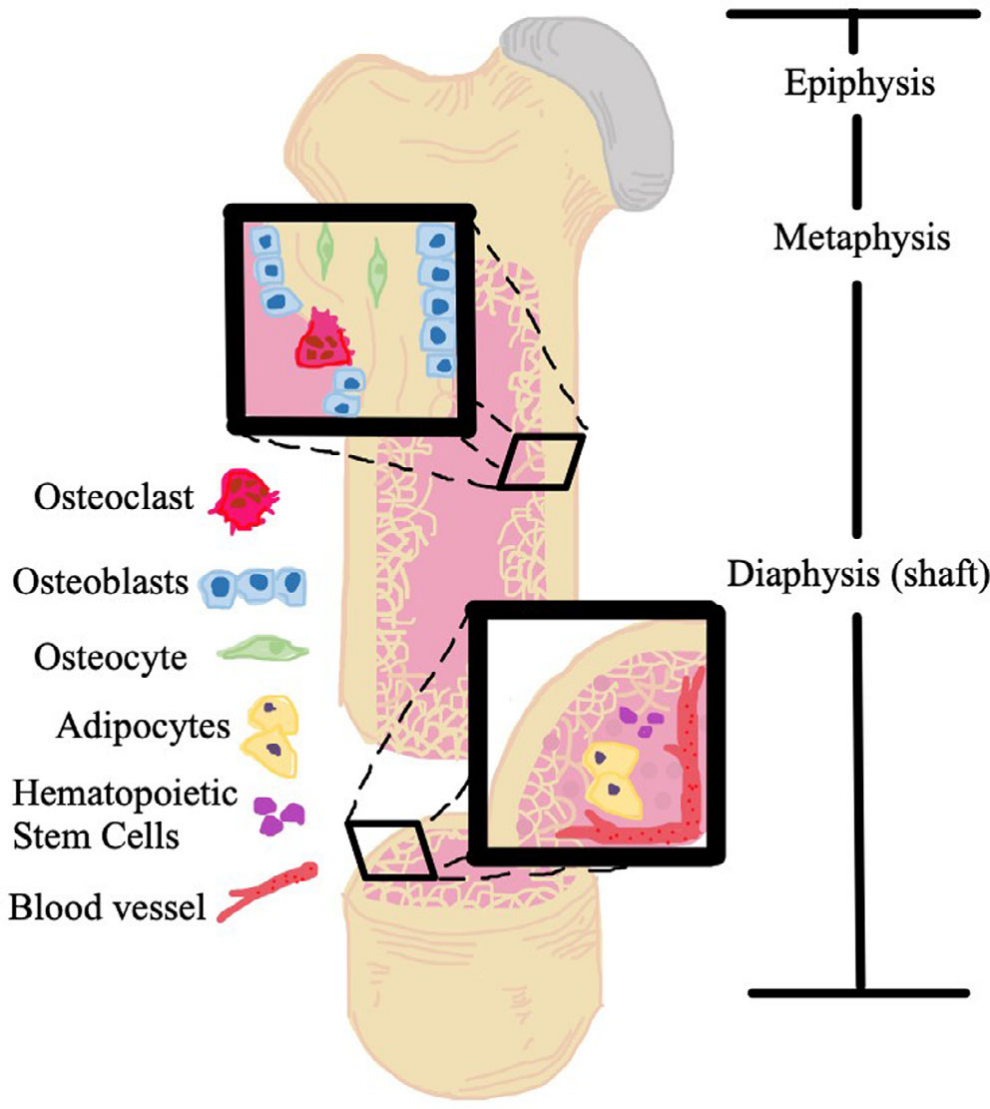
Artistic rendition of bone structure and bone marrow niche, including osteoblasts, osteoclasts, osteocytes, bone marrow adipocytes, and hematopoietic stem cells.

Histologically, bone can be categorized into two distinct tissue types: cortical and trabecular. Cortical bone (80% of skeletal mass)^1^, ^2^ makes up the outer layer and is more readily found within the diaphysis (Fig. 1). Conversely, the trabecular bone (20% of skeletal mass) occupies the inner space and is mostly localized to the metaphysis and epiphysis (Fig. 1).^3^ On the microscopic level, bone is composed of cells, such as osteocytes, osteoprogenitor cells, osteoblasts (bone‐forming cells), and osteoclasts (bone‐resorbing cells), and extracellular matrix (ECM). The ECM can be further separated into organic components (30% by weight), including proteoglycans, glycosaminoglycans, glycoproteins, osteonectin (which anchors bone mineral to collagen), and osteocalcin (calcium‐binding protein), as well as inorganic components (60% by weight) and water.^1^ A calcium phosphate mineral termed hydroxyapatite is the major inorganic component of bone.^4^ The mineral‐based construction of bone requires that all the components necessary to build strong bone be incorporated in proper portions and locations, and with proper connections with other organic matrix components and cellular components of bone.

The dynamic nature of bone creates a constant state of remodeling and contributes to a unique microenvironment. Within the bone marrow (BM), osteocytes are mechanosensitive cells that influence and balance the activity between osteoclasts and osteoblasts. Homeostasis is maintained via an intricate balance between osteoclasts and osteoblasts throughout their lifecycle along the surface of trabecular bone and the endocortical side of cortical bone. Periosteal osteoblasts and some osteoclasts line the periosteum of bones and interface with muscle cells outside of bone. In the BM space, immune cells derived from the hematopoietic lineage are also in close proximity to the bone surface, creating stem cell niches and microenvironments.^5^, ^6^, ^7^ Vascular niches are found within the BM where endothelial cells and pericytes create sinusoidal, leaky vessels that allow for easy passage of cells between the BM and the circulatory system.^8^ Furthermore, BM adipocytes also share this space and play an important role in health and disease progression.^9^ It is now widely accepted that the number of adipocytes increases with age and in response to various induction signals. The increased number of BM adipocytes is often correlated with reduced bone mass, and has been implicated in negatively regulating hematopoiesis,^10^, ^11^ although they also have been shown to support hematopoiesis through the production of stem cell factor.^12^ Not only are BM adipocytes involved in regulating the local BM environment, but they may influence overall body homeostasis.^13^, ^14^

When cancer cells develop in or metastasize to the BM from a distant site via blood vessels, not only do they enjoy an abundance of growth factors, but they also have opportunities to interact with mesenchymal and hematopoietic progenitors in various stages of differentiation. In essence, the BM provides the cancer cells, as well as healthy hematopoietic cells, a favorable environment for survival during neoplastic processes or during BM re‐establishment, in the case of a BM transplant. Numerous environment factors—biochemical properties such as hypoxia and chemokine gradients, as well as cellular composition—maximize the chances for metastatic cancer cell survival and colonization in their new environment.^15^, ^16^, ^17^, ^18^

### The historical transition from 2D to 3D in vitro models

Over a century ago, attempts to grow cells and tissues outside the body started by Ross Harrison^19^ with the “hanging drop technique,” in which a small tissue sample was grown in medium. Along the way, advances were made by Alexis Carel and Charles Lindbergh, who developed techniques to keep tissues continuously growing on glass plates, and the discovery of HeLa cells in the 1960s and 1970s led to methods for the clonal growth of cells.^20^ In attempts to create a cell culture environment that resembles the human body more closely, 3D models were introduced by Hamburger and Salmon in the 1970s using a soft agar solution.^21^ Despite the introduction of the floating gel concept in the 1970s, it is believed that the term “three‐dimensional culture model” was coined with the assays developed by Barcellos‐Hoff and colleagues and Petersen et al. in 1989 and 1992, respectively.^22^, ^23^, ^24^

Typically, 2D in vitro models, in which cells are cultured on a single layer on a plastic plate in a controlled environment similar to the human body, have been in wide use in the pharmaceutical industry.^25^ These models do not mimic the natural environment of tumor growth in the sense that cancer cells grow on a single layer, nearly half of the cells in direct contact with the surface of a growth plate. Naturally, these cells lose certain characteristics, such as signaling pathways, which would alter the cells' metabolism, growth, and differentiation to adapt to a new environment.^26^, ^27^, ^28^, ^29^, ^30^ Furthermore, traditional 2D cancer cell cultures do not realistically recapitulate tumor heterogeneity.^31^ Tumor development is a multistep process, and a series of factors, such as phenotypical heterogeneity, biological context, and heterotypic crosstalk, as well as the microenvironment influence survival and proliferation.^32^ Although the 2D models cannot offer these requirements, they do offer some advantages in regard to ease of maintenance, simplicity, and cost effectiveness compared with other tissue culture models, such as 3D and animal models.^33^ To mimic human cancer growth, in vivo animal models are also widely used to study cancer cells. Animal models can provide a complex physiologic structure that is reasonably similar to the human cell and organ system.^34^, ^35^, ^36^, ^37^

Although animal models can be adequately controlled and modified for pharmaceutical studies and discoveries, there are still challenges, such as species’ differences, the high costs of animal model maintenance, and potential ethical issues.^30^ According to a review by Alemany‐Ribes and Semino, it is estimated to take 10 to 12 years to develop a new cancer drug, and only 5% of new drug applications receive approval from the U.S. Food and Drug Administration.^38^ In another study that analyzed 10 new drugs from 10 pharmaceutical companies, the median time for drug development was 7.3 years (range 5.8 to 15.2 years) at a median cost of $648 million (varying from $157 million to $1.950 billion).^39^ Ranga and colleagues point out that part of the reason the development costs and time are inflated is that promising potential drugs often underperform in vivo compared with the expectations they suggested from in vitro studies. Currently, most primary drug studies and screens are performed on cell lines grown using 2D methods, and are required to have at least two successful animal models prior to proceeding to human clinical trials.^40^ Because of the high rate of failure from 2D to animal models and from animal to human studies, 3D cultures may represent an excellent option to reduce the amount of time and resources required to transition a discovery from the laboratory to patient care.^38^

Since the development of the first 3D models,^22^, ^23^, ^24^ newer in vitro 3D cell culture systems, which simulate in vivo physiology and tissue microenvironment, have been developed and now play a crucial role in research. 3D models have been shown to recreate the natural microenvironment of tumor cells more accurately than 2D models in a relatively simple and cost‐effective way,^32^, ^41^, ^42^, ^43^ and have been utilized in preclinical evaluations to test efficacy and safety profiles of potential pharmacotherapeutics against cancer.^44^ Cancer cells that proliferate in 3D culture systems display cell–cell and cell–matrix interactions, gene expression levels, signaling pathways, and structural integrity, which are more comparable to in vivo tumors than 2D models are.^45^ Furthermore, 3D cultures demonstrate the features of solid tumors, including physiological responses and drug metabolism, more accurately than 2D systems can.^46^

In general, tumors are poorly vascularized; cancer cells need to adapt to survive in hypoxia and other unfavorable metabolic environments. As oxygen and nutrients become scarce, cancer cells (especially those that are farther from vascular sources) manage to stay alive by slowing their cell cycle or by becoming dormant.^47^ Hypoxia has been known to induce drug resistance, and 3D culture systems—as opposed to 2D—can recapitulate the oxygen gradient that is observed in in vivo tumors.^48^ However, 3D culture systems also have shortcomings. When compared with 2D cultures, 3D cultures require more time and money to develop and maintain. Also, in many 3D models, the efficiency, ease, and reproducibility of work have been shown to be inferior to 2D models.^49^, ^50^ Although 2D, 3D, and animal models all have their benefits and drawbacks, 3D culture methods are considered to reduce the transitional gap between in vitro *and* in vivo research. Still, Hoarau‐Véchot and colleagues argue that it is not possible to clearly determine the general advantages and limitations of 3D compared with 2D models because of the wide range of techniques that are available.^51^

### Applications of 3D models for organs, tissues, and tumor microenvironments

Once cancer cells metastasize to bone, the prognosis is generally poor. In addition, the cost of care, both financially and physically, is burdensome on patients and their families. For a patient with a neoplastic bone disease, such as multiple myeloma (MM) or bone‐metastatic breast or prostate cancer (PCa), complications, including bone pain, fractures, hypercalcemia, spinal cord compression, and more, are typically encountered as the disease progresses. If a patient manages to endure the challenges of treatment, the expected survival time varies greatly depending on the original cancer type; for instance, the timeline is measured in months for lung cancer and in years for prostate and breast cancer.^52^ Of course, these consequences negatively affect a patient's quality of life and lifespan.^53^, ^54^ Nevertheless, despite the positive changes in treatment and prognosis for patients with bone metastasis as a result of multidisciplinary treatment options,^55^ the quest for yet a better therapy regimen is being undertaken worldwide to maximize survival rates and further improve quality of life.

In the pursuit of this quest, many researchers seek to narrow the gap between 2D models and clinical trials using 3D models, which can often simulate the natural environment of tumor cells very well. For instance, signaling pathways and drug metabolism can be replicated more precisely in a 3D model,^38^ possibly minimizing time and resources invested in potential drugs that fail to materialize. Furthermore, 3D models can be used to enhance our understanding of mechanisms that lead to drug resistance, which can be further exploited for drug development.^38^, ^56^ The latest advancements in successful 3D models, such as newer synthetic biomaterials and better‐tuned mechanical and chemical features, are helping to fight against cancer and are expected to result in better systems for preclinical research, early drug screening, and even earlier cancer diagnosis in the future.^38^

## Bone cancer applications of 3D Bone models

Challenges in treating malignant bone disease result, in part, from the shelter that the bone and BM provide from the immune system, and chemotherapeutic and radiotherapeutic agents to the point that the BM microenvironment provides de novo resistance.^57^, ^58^ Thus, it is critical to simulate this interaction between tumor cells and the in vivo microenvironment with in vitro studies to most accurately ascertain the clinical significance of any experimental observations and design better therapies to overcome this BM‐driven drug resistance. An overview of materials currently being used for in vitro 3D models of bone cancer is provided in Table 1. In this review, we discuss the use of 3D models in two primary BM cancers: MM and leukemia.

**Table 1:** 3D Models for the Biology of Blood and Cancer

Biomaterial	Scaffold type	Cancer	Refs
Natural materials			
Silk fibroin	Scaffold, hydrogel, mat	MM, prostate, breast, osteosarcoma	59, 60, 61, 62
Collagen	Hydrogel, scaffold	Breast, prostate, osteosarcoma	63, 64, 65
Chitosan‐alginate	Scaffold	Prostate, glioblastoma, hepatocellular carcinoma	66, 67
Hyaluronic acid	Scaffold, hydrogel	Renal cell carcinoma, MM	68, 69
Bacterial nanocellulose	Scaffold, hydrogel	Neuroblastoma, osteosarcoma, prostate, renal cancer, breast	70, 71
Native ECM	Scaffold	MM, breast	72, 73
ECM/cartilaginous matrix/Matrigel	Scaffold, hydrogel	Breast	74
Chitosan (with or without HA or collagen)	Scaffold	Breast	75
Cell sheets over medical‐grade polycaprolactone‐tricalcium phosphate	scaffold	prostate	76
Synthetic materials			
Poly(ethylene) glycol	Hydrogel	Breast, prostate	77, 78, 79
Poly(ε‐caprolactone)	Hydrogel	Breast, prostate, osteosarcoma, Ewing sarcoma	80, 81, 82
Poly(amino acid‐)‐based polymers	Hydrogel	Osteosarcoma	83
PLG (nonmineralized) and PLG mineralized with HA	Scaffold	Breast	84

Adapted with permission from Sitarski and colleagues.^33^

BM = bone marrow; ECM = extracellular matrix; HA = hydroxyapatite; MM = multiple myeloma; PLG = poly(lactide‐co‐glycolide).

### Multiple myeloma

The BM niche is a complex environment that allows for interactions between mesenchymal stem cells (MSCs), osteoblasts, osteoclasts, and BM adipose tissue. The relationships between cells in the BM niche are understood to varying degrees, but it is clear that interactions between niche members are implicit in the progression of various disease processes. Malignant neoplasms are one such group of diseases and their effective study requires further understanding of this multifaceted niche.

**Figure 2:**
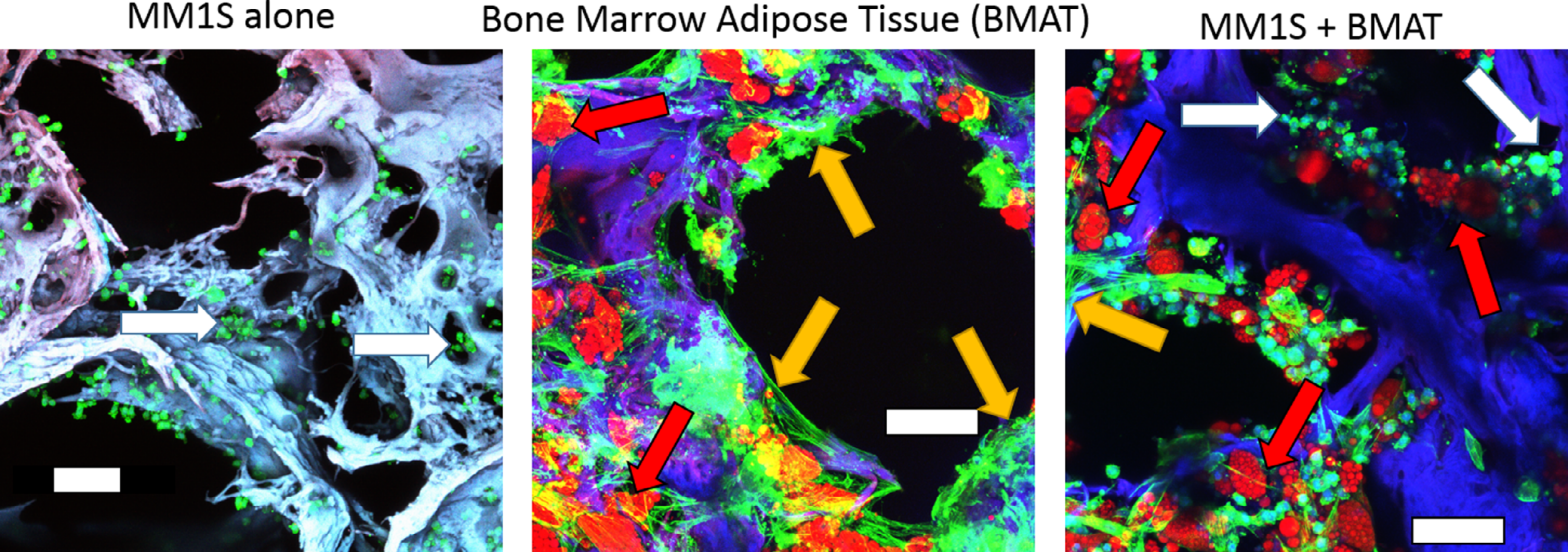
Example of 3D human bone marrow adipose tissue (hBMAT) coculture with MM1S myeloma cells. Maximum projections of confocal imaging of cocultures. hBMAT was seeded to silk, cultured until confluent, and switched to adipogenic media for 37 days. Then scaffolds were switched to a coculture media and seeded with GFP+/Luc + MM1S cells and imaged at 1 week. Fixed scaffolds were stained with oil red O (lipids = red), phalloidin (actin = green), and 4,6‐diamidino‐2‐phenylindole (nuclei = blue). Scaffold is autofluorescent (blue). Both adipocytes and undifferentiated stromal cells are observed throughout the BMAT. Scale bar = 100 μm. White arrows indicate tumor cells; yellow arrows indicate stromal cells; red arrows indicate adipocytes.

The use of 3D models in primary malignancies of the bone and BM has yielded great insight into these disease processes and is currently paving the way toward improved therapeutics and prognoses. MM, a cancer caused by clonal expansion of mutant plasma cells in the BM, represents 10% of hematological malignancies and comprised 2% of all cancer deaths in 2017.^85^ MM is recognized as an incurable malignancy with death most commonly resulting from relapsed refractory disease. The employment of 3D models in myeloma research has elucidated novel means of studying the interactions between tumor and the microenvironment, as well as mechanisms of drug delivery and resistance. To simulate MM in the BM microenvironment, Jakubikova and colleagues^86^ used a hydrogel 3D model that included MSC in coculture with primary BM cells from MM patients. Using this system, they have shown integrins, ECM molecules, N‐cadherin, and CXCL12 by MM patient‐derived MSCs in 3D. Osteoblastogenic differentiation and clinical resistance to novel and conventional chemotherapeutics were confirmed in this hydrogel model.^86^ The BM adipocyte has recently been named a key player in the progression of myeloma. Fairfield and colleagues^87^ have instituted a 3D silk scaffold on which they have successfully cultured BM adipocytes for 3 months and cocultured BM adipocytes with myeloma cells for 2 weeks (Fig. 2). As compared with 2D cultures, general metabolic, DNA replication, ribosome, and proliferation pathways were upregulated in BM adipocytes in this model.^87^ Implementation of this silk fibroin scaffold by Reagan and colleagues to study myeloma growth, osteogenesis inhibition, and MM–MSC interaction unveiled myeloma support of endothelial cell assembly into capillary‐like structures and cell adhesion‐mediated drug resistance.^59^

Using a 3D Matrigel environment to recapitulate the BM niche, the tumoricidal potential of TEGs (αβT cells engineered to express a defined γδTCR), novel αβT cells engineered to express a defined γδTCR cell receptor (TCR), was examined and compared with a conventional 2D model. It was found that the 3D model enabled superior analysis of the homing behavior of the TEGs, in addition to improved evaluation of the on‐ and off‐target effects of this potential therapy.^88^ Braham and colleagues further employed this Matrigel model to examine liposomal drug delivery in the context of myeloma, which showed increased cytotoxic effects of bortezomib and doxorubicin as compared with free drugs.^89^ To study oxygen and drug gradients, drug resistance, and myeloma–microenvironment interaction, de la Puente and colleagues created 3D tissue‐engineered BM cultures derived from BM supernatant of MM patients. Their study found that this 3D model more accurately simulated the tumor interaction with the microenvironment, and that drug resistance was induced at a higher rate than in 2D or commercial 3D systems.^72^ Zhang and colleagues^90^ have implemented another innovative in vitro model comprised of ossified 3D tissue in combination with an eight‐chamber microfluid culture device that has facilitated the successful seeding of mononuclear cells of BM aspirates from MM patients. Their results showed mononuclear cell homing to the osteoblast layer and expansion of the mononuclear cells over a 3‐week period. This is a promising model for the development of personalized therapies, cost‐effective drug development, and the study of resistance and relapse as it more realistically models perfusion‐related mass transport and shear stress present in vivo, in addition to having the capacity to use patient BM aspirates.^90^ Through the use of diverse 3D scaffolds, researchers studying myeloma have proven the efficacy, necessity, and value of implementing 3D models in preclinical research.

### Leukemia

Leukemia is a group of hematologic malignancies that develop from the dysregulated growth of BM progenitor cells of myeloid or lymphoid origin. Although these malignancies are of hematologic origin, their development in and destruction of BM make accurate models of the BM niche essential in the study of its biology and development of therapeutic interventions. Comparison of a traditional 2D model of cells in suspension with a 3D model, utilizing copolymer disks for the culture of acute myelogenous leukemia (AML) cells with or without expanded human MSCs, was done to distinguish differences in response to the chemotherapeutics doxorubicin and cytarabine. Successful penetration of and unobstructed flow through the scaffold by the chemotherapeutics and media components were confirmed via confocal microscopy and steady‐state fluorescence. This study showed increased resistance to chemotherapeutic‐induced cell death in the 3D model as compared with the 2D model, which was hypothesized to be related to the expression of N‐cadherin in the 3D coculture system.^91^ In a study of treatment‐resistant chronic myelogenous leukemia lacking a BCR‐ABL1 kinase domain, a poly(methylmethacrylate)‐hydroxyapatite (PMMA‐HA) fiber scaffold generated by pressurized gyration was employed in an effort to establish a system that replicates clinically observed refractory disease. Compared with 2D models, this 3D model showed the relative resistance of cancer cells to imatinib and doxorubicin, which makes it a more accurate platform for further investigation into leukemic disease and drug resistance.^92^ Decellularized Wharton's jelly matrix (DWJM) has also been used as scaffolding on which to conduct 3D in vitro leukemia studies. Wharton's jelly is the gelatinous component of the umbilical cord; it contains collagen, fibronectin, lumican, and hyaluronic acid, all of which are constituents of the BM ECM. This model of the BM microenvironment led to decreased proliferation and differentiation of the leukemia cells, as well as changes in cellular morphology such that the cells had a more spindle‐shaped appearance. When cultured with doxorubicin, leukemia cells in the DWJM had lower levels of apoptosis and greater colony‐forming potential compared with 2D cell‐suspension models. Again, this finding is associated with increased expression of N‐cadherin in the 3D model.^93^ A 3D polyurethane model to study AML has been established by Blanco and colleagues.^94^ This model has been used to study the effects of physiologic levels of oxidative and glucose stress of AML and has shown that cells grown in 3D are better able to adapt to oxidative stress than those cells grown in a 2D model. Glucose was found to have a greater impact on cell growth in the 3D model. These observations could be used to facilitate treatment optimization.^95^

### Bone‐metastatic breast cancer

Along with primary BM cancer, metastatic disease comprises a significant portion of malignancy involving the marrow, often with greater morbidity and mortality. Of the cancers that commonly metastasize to bone, here we will focus on breast cancer and PCa, which comprise 80% of bone metastatic disease.^96^ Bone metastases occur in up to 40% of patients with breast cancer and are found in the red marrow of the axial skeleton.^6^, ^97^ The red marrow is actively hematopoietic and requires a substantial blood flow, allowing cancer cells access to the environment through the porous bone matrix and capillary sinusoids. Once there, adhesive molecules on tumor cells interact with cells on the BM niche and begin colonization.^6^, ^98^ To better study this disease process, Bray and colleagues^99^ generated a 3D biphasic microenvironment of breast tumor tissue, spatially separated by a mineralized bone construct of human primary osteoblasts in a cryogel, which proved superior to the established 2D and cell‐suspension models. Furthermore, this model allowed the study and manipulation of migration and interaction patterns of cancer cells. Additionally, it is a dynamic platform that can be altered to study cell response and function. With support of normal in vivo cellular functions, such as the use of adhesion peptides and controlled release of signaling molecules, this model more accurately recapitulates the tumor microenvironment.^99^ The use of a novel 3D microfluidic model was similarly employed to study the transendothelial migration of metastatic breast cancer cells and their interactions within bone matrix. Use of this model highlighted the role of breast cancer cell receptor CXCR2 and bone‐secreted chemokine CXCL5 in extravasation to the BM.^100^ Salamanna and colleagues^97^ aimed to uncover the conditions that make bone a desirable place for cancer growth. Using a humanized 3D model derived from human bone tissue from healthy and osteoporotic patients, an in vitro simulation of pre‐ and postmenopausal bone was created. Here, they found that cancer cells preferentially colonized the osteoporotic bone, which mimicked the effects of decreased estrogen in the postmenopausal bone.^97^ It is known that mammary tissue is capable of calcification and that it possesses osteomimetic properties. To study these properties in relation to bone metastasis, a novel 3D collagen glycosaminoglycan scaffold was established; this was shown to support the growth and mineralization of breast adenocarcinoma cells.^101^ Colonization and degradation of osteoblastic tissue, cancer‐bone adhesion, and tissue penetration by metastatic breast cancer cells was shown by Dhurjati et al.^102^ This group created a 3D model of mineralized osteoblastic tissue grown from isolated osteoblasts in a specialized bioreactor in an effort to mimic the BM microenvironment.^102^ Morphologic changes in native members of the BM niche, such as the osteoblast, were observed when cultured in 3D with metastatic breast cancer cells. In this model, the native cells took on a spindle‐shaped morphology and aligned parallel to cancer cells. As compared with the 2D model, the proliferation of the cancer cells in this 3D specialized bioreactor system was more similar to that observed clinically.^103^

### Bone‐metastatic prostate cancer

In advanced PCa, 70% of patients have metastatic bone disease. PCa cells are able to metastasize to the BM in an analogous way to breast cancer cells in that they take advantage of the high blood flow to the BM and the stasis of blood at the BM sinusoids, which allow the cells to extravasate and colonize.^96^ Bone metastatic PCa is distinct from other cancers in the BM, in that it is osteoblastic rather than osteolytic.^104^ To improve the morbidity and mortality rates for metastatic PCa patients, 3D models are being used to study the disease process. To recreate this environment and incorporate key players such as endothelial cells and osteoblasts in addition to the PCa cells, a two‐layer microfluidic system to culture 3D multicell‐type spheroids was developed. Testing of this model showed tumor cells retained viability, but decreased proliferation activity in a fashion that more accurately resembles the in vivo experience.^105^ Choudhary and colleagues used a 3D network of primary human osteocytes to create an engineered bone tissue model to study the interaction of PCa cells with bone. Unlike 2D cultures, this 3D model showed an increase in expression of fibroblast growth factor 23 and alkaline phosphatase, accurately depicting the bone niche observed clinically in metastatic PCa.^106^ The use of polymer‐nanoclay‐based 3D scaffolds for the growth and study of PCa cells in coculture with MSCs yielded the formation of tumoroids with tight cellular junctions and hypoxic cores. The hypoxia was found to increase angiogenesis. MSCs in this model were found to differentiate into mature osteoblasts and showed enhanced mesenchymal to epithelial transition and inhibited epithelial to mesenchymal transition. In coculture, the PCa cells increased osteoblastic gene expression. These findings were in line with clinical observations making this model ideal for studying tumor biology and drug development for osteoblastic cancers.^107^, ^108^ A Matrigel culture system was used to observe the coculture of PCa cells with bone stromal cells and prostate epithelial cells. This model showed an upregulation in factors essential to metastatic dissemination. These factors included epithelial‐to‐mesenchymal and chemokine protein constituents, N‐cadherin, and CXCR7. The N‐cadherin expression was determined to be modulated by PCa cell‐secreted soluble factors, whereas CXCR7 expression was caused by cell‐to‐cell interaction.^109^ Fitzgerald and colleagues^34^ created three types of collagen‐based scaffolds containing either collagen alone or in combination with glycosaminoglycan or nanohydroxyapatite. This model was used to examine PCa proliferation, viability, prostate‐specific antigen (PSA) secretion, matrix metalloproteinase (MMP) secretion, chemosensitivity to docetaxel, and nanoparticle gene delivery. PCa cells grown in this model were found to secrete elevated PSA and decreased MMP. They found that PCa cells infiltrated the scaffolds and exhibited more chemoresistance in 3D as compared with 2D. Cellular uptake of the siRNA nanoparticles was achieved and exhibited successful gene knockdown. This model is promising in the testing and development of PCa therapies that can infiltrate the bone microenvironment.^34^

The use of 3D models to study primary and secondary malignancies of BM has proven efficacious in allowing more accurate simulation of the in vivo condition. Understanding of the underlying biology of these diseases has been improved by these models. Additionally, observations of drug efficacy in these models have more closely resembled patients’ experiences and have provided an improved model for drug development and testing.

## Discussion

In the century since Ross Harrison pioneered the study of cells outside the body, the development of new medications and other technological advances in medicine have helped extend life expectancy from 47 to 78 years.^110^, ^111^ Furthermore, in the past two decades, 5‐year cancer survival rates are up 39% across all cancer types.^111^ More than 1800 cancer medications are in development; 80% is considered to be first‐in‐class. Many patients will benefit from the new research studies and drugs that are coming to the market, and will enjoy a better quality of life. However, it is important to be aware of the costs related to the development of a new drug. Figures vary from one study to another, but estimates from the Tufts Center for the Study of Drug Development (Boston, MA, USA) posit that a new drug comes at a cost of $2.6 billion and 10 years’ worth of development time.^110^ Between 1998 and 2014, 10 new lung cancer drugs were introduced, but 167 others were deemed unsuccessful pharmaceutic endeavors.^110^ As previously mentioned, the conventional approach is from 2D models, to animal studies, then to human clinical trials. However, 2D models do not accurately predict drug effectiveness, pharmacokinetics, or pharmacodynamics. Simultaneously, animal models are limited in forecasting how drugs will behave in the human body during clinical trials because of unique characteristics that exist in different species and the high cost of in vivo experiments, relative to in vitro experiments. In light of the high failure rate and the astronomical costs to introduce a new drug, 3D models can potentially provide a pathway to maximize efficiency and expedite the drug discovery process. There is mounting evidence that in vitro 3D models can be useful in improving preclinical research and utilized in achieving decisions earlier during drug development.^112^ By eliminating compounds that would have limited clinical applicability and modifying approaches before reaching animal models or clinical trials, researchers can decrease the time and financial resources invested in unsuccessful drugs.

Additionally, 3D models can be a conduit to more personalized medicine. For many patients, a standardized pharmaceutical regimen can be sufficient in treating cancer. However, tumor heterogeneity still remains a major obstacle in cancer therapy.^112^, ^113^ 3D models can be used with cancer cells extracted from patients to understand how the tumor would respond to drug therapy, potentially saving time and money for patients to arrive at the optimal medication and dosage.

There are still disadvantages that need to be addressed before 3D models can be adopted more readily. There have been reports of inconsistencies in 3D cultures and duplicability of results.^49^, ^50^, ^114^ Furthermore, 3D models are more difficult to handle, which may require a higher level of training and added expense, and utilize analytic programs that can process larger and more complex data.^114^ More studies might be indicated before we see a significant increase in researchers adopting 3D models as part of a standard investigation. However, the involvement of the microenvironment in understanding tumor progression and metastasis is expected to grow in the future,^115^ at which point we may finally see the rise of finetuned 3D models that can fill the gap in current cancer research.

## Disclosures

The authors have no conflicts of interest.
